# Impact of Prebiopsy Multiparametric Magnetic Resonance Imaging on Prostate Cancer Detection in Switzerland

**DOI:** 10.1016/j.euros.2025.01.004

**Published:** 2025-01-24

**Authors:** Thomas Paul Scherer, Dominik Menges, Uwe Bieri, Lea Wildisen, Katharina Staehelin, Daniel Eberli, Sabine Rohrmann, Cédric Poyet

**Affiliations:** a Department of Urology University Hospital Zürich (USZ) University of Zürich (UZH) Zürich Switzerland; b Epidemiology Biostatistics and Prevention Institute University of Zürich (UZH) Zürich Switzerland; c Department of Medical Epidemiology and Biostatistics (MEB) Karolinska Institutet (KI) Stockholm Sweden; d Department of Surgery Division of Urology Cantonal Hospital Baden Aargau Switzerland; e National Institute for Cancer Epidemiology and Registration (NICER) Zürich Switzerland; f National Agency for Cancer Registration (NACR) Zürich Switzerland; g Cancer registry of the Cantons Zürich Zug Schaffhausen and Schwyz University of Zürich Zürich Switzerland; h Department of Urology City Hospital Triemli of Zürich Zürich Switzerland

**Keywords:** Multiparametric magnetic resonance imaging, Prostate cancer, Biopsy, International Society of Urological Pathology grade group

## Abstract

**Background and objective:**

Multiparametric magnetic resonance imaging (mpMRI)-guided prostate biopsies have become the standard of care. This study aims to analyze changes in the distribution of detected prostate cancer (PCa) risk groups in Switzerland during the adoption period of a multiparametric magnetic resonance imaging (mpMRI)-guided prostate biopsy.

**Methods:**

This ecological study analyzed prostate biopsies from a tertiary hospital and PCa diagnoses from the National Agency for Cancer Registration in Switzerland between January 2005 and December 2019. A survey assessed mpMRI uptake in Swiss urological centers. PCa risk group proportions were calculated and compared for the entire period and annually.

**Key findings and limitations:**

A total of 4890 biopsies in the hospital dataset and 74 747 national PCa cases were analyzed. Before mpMRI availability, 72.6% of hospital biopsies were PCa negative, with detected cases including 46.4% low risk (LR), 30.5% intermediate risk (IR), and 23.2% high risk (HR). After the availability of mpMRI, 45.7% were PCa negative, with 24.6% LR, 49.0% IR, and 26.5% HR. National mpMRI uptake began in 2008, surpassing 95% by 2019. In 2005, 3448 PCa cases were recorded across 14 cantons, with 46.3% LR, 33.1% IR, and 20.6% HR. By 2019, 6868 cases were registered in 23 cantons, with 28.0% LR, 48.9% IR, and 23.2% HR.

**Conclusions and clinical implications:**

After implementation of mpMRI, fewer negative prostate biopsy results were observed. Furthermore, a shift was detected in the distribution of risk groups, with an increase in the proportion of IR cases and a decline in LR cases alongside the uptake of mpMRI. The proportion of HR cases remained essentially constant over time. Further research is needed to determine whether this reflects improved stratification or an artifact of the changed diagnostic pathway.

**Patient summary:**

In this study, we examined how the detected prostate cancer grades changed during the adoption of multiparametric magnetic resonance imaging (mpMRI)-guided prostate biopsies in Switzerland between 2005 and 2019. After mpMRI, fewer negative biopsies occurred and the distribution of prostate cancer grades changed, with more intermediate-risk and fewer low-risk cancers identified, while high-risk cases remained stable.

## Take Home Message

Uptake of multiparametric magnetic resonance imaging–guided biopsies reduced negative biopsies and altered the distribution of prostate cancer grades, with more intermediate-risk and fewer low-risk cases. Further research is needed to determine whether this reflects improved stratification or an artifact of the changed diagnostic pathway.

## 1 Introduction

Prostate biopsy practice has evolved over the past decade. According to international guidelines, multiparametric magnetic resonance imaging (mpMRI)-guided biopsies have become the standard of care, replacing conventional transrectal ultrasound (TRUS)-guided biopsies [1], [2]. These changes in recommendations arose because randomized controlled trials demonstrated that using mpMRI before prostate biopsies resulted in a higher detection rate of International Society of Urological Pathology (ISUP) grade group ≥2 prostate cancer (PCa) cases, whereas fewer cases of ISUP grade group 1 cases were detected [3], [4]. Furthermore, mpMRI before biopsies reduced the frequency of PCa-negative prostate biopsy results [3], [5]. However, real-word data on the impact of an mpMRI-guided biopsy are still sparse. To understand the impact of mpMRI-guided biopsies in comparison with the conventional TRUS-guided biopsies, we aimed to investigate the distribution of diagnosed risk groups at both the hospital and the population level during the period when the mpMRI-guided biopsy became the dominant biopsy technique.

## 2 Patients and methods

To assess the development of PCa risk groups at diagnosis at the hospital level, all prostate biopsies between January 2005 and December 2019 at our tertiary care academic center were reviewed retrospectively. Owing to uncertainties about the impact of the COVID-19 pandemic on PCa detection, we limited our analysis to data up to the end of 2019. Age, year of diagnosis, prostate-specific antigen (PSA), biopsy method, Gleason score (GS), and clinical T stage were recorded. PCa was divided into the following risk groups: low-risk (LR) PCa was defined as ISUP grade group 1, intermediate-risk (IR) PCa included ISUP grade groups 2 and 3, and high-risk (HR) PCa contained ISUP grade groups 4 and 5 [6].

To assess national population trends in the incidence of PCa prognostic risk groups, data from cantonal cancer registries provided by the Swiss National Agency for Cancer Registration (NACR) of the same period (from January 2005 until December 2019) were utilized.

The following patient characteristics were exported for each year of the study period: age (in 5-yr intervals), PSA value, GS, TNM stage at diagnosis, and cantonal registry. Furthermore, annual age-standardized incidence rates of PCa were calculated. Since cancer registration in Switzerland has been mandatory only since 2020, some cantons were not covered by cancer registration during the whole study period. Age standardization was done using the European standard population 2013 [7]. Swiss cancer registries do not collect information on the biopsy method. Hence, we conducted a survey to estimate and assess the uptake of mpMRI-guided biopsies in Switzerland over the years. It was sent via a secured e-mail to each head of the Department of Urology in any of the 62 Swiss hospitals belonging to the three largest size categories, that is, having >6000 inpatient cases per year. The survey assessed for each department whether mpMRI is conducted prior to the biopsy, when mpMRI was first implemented and its frequency, and the number of biopsies conducted per year (see Supplementary Tables 1–3).

The distribution of mpMRI uptake and PCa risk groups in Switzerland were analyzed descriptively and displayed graphically for each year. Wald 95% confidence intervals (CIs) were calculated for proportions of PCa risk groups. Additionally, subgroup analyses were conducted for each cantonal cancer registry that received more than one survey answer, had a survey response rate of at least 60%, and completed cancer registration for the study period. Spearman correlation coefficients between mpMRI usage and the frequency of risk groups over the years were calculated for the whole study population and for regional subgroups. Furthermore, the influence of missing data on the distribution of risk groups was evaluated in a sensitivity analysis that excluded data from cantonal cancer registries, with missing tumor grade information exceeding 50% and 25%, respectively.

Data entry for the hospital dataset was done using Microsoft Access (version 2016; Microsoft Corporation, Redmond, WA, USA). A statistical analysis was performed using STATA 18 (StataCorp., College Station, TX, USA). The study plan was reviewed and approved by the local ethics committee (KEK Nr. 2023-01149).

## 3 Results

Between 2005 and 2019, a total of 4890 prostate biopsies were performed in the hospital group. The men of the hospital dataset underwent a prostate biopsy at a median age of 64.7 yr (interquartile range [IQR]: 59.1–69.7 yr) with a median PSA value of 5.5 ng/ml (IQR: 3.6–8.9 ng/ml).

In the NACR dataset, a total of 74 747 of PCa cases were documented. During the study period, registry coverage increased from 58.6% of the Swiss population to 87.9%. The clinical characteristics of both datasets are illustrated in Table 1.

**Table 1:** Clinical characteristics of men undergoing prostate biopsy in the hospital dataset and of prostate cancer patients in the National Agency for Cancer Registration dataset between 2005 and 2019

	Negative prostate biopsies in the hospital data	Prostate cancer diagnoses in the hospital data	Prostate cancer diagnoses in the National Agency for Cancer Registration dataset
Number	2840	2050	74 747
<50 yr, *n* (%)	88 (3.1)	22 (1.1)	707 (0.9)
50–54 yr, *n* (%)	201 (7.1)	109 (5.3)	2615 (3.5)
55–59 yr, *n* (%)	543 (19.1)	241 (11.8)	6455 (8.6)
60–64 yr, *n* (%)	708 (24.9)	349 (17.0)	11 927 (16.0)
65–69 yr, *n* (%)	727 (25.6)	541 (26.4)	15 716 (21.0)
70–74 yr, *n* (%)	430 (15.1)	473 (23.1)	15 168 (20.3)
75–79 yr, *n* (%)	120 (4.2)	217 (10.6)	10 082 (13.5)
80–84 yr, *n* (%)	22 (0.8)	62 (3.0)	6750 (9.0)
85+ yr, *n* (%)	1 (<1)	36 (1.8)	5327 (7.1)
PSA (ng/ml), median (IQR)	5.0 (3.3, 7.7) *n* = 2785	6.4 (4.1, 10.9) *n* = 1986	9.3 (5.9, 23.6) *n* = 51 673
Pr. Vol. (ml), median(IQR)	43.0 (30.0, 60.0) *n* = 2342	40.0 (30.0, 51.0) *n* = 1716	–

IQR = interquartile range; Pr. Vol. = prostate volume; PSA = prostate-specific antigen.

**Fig. 1:**
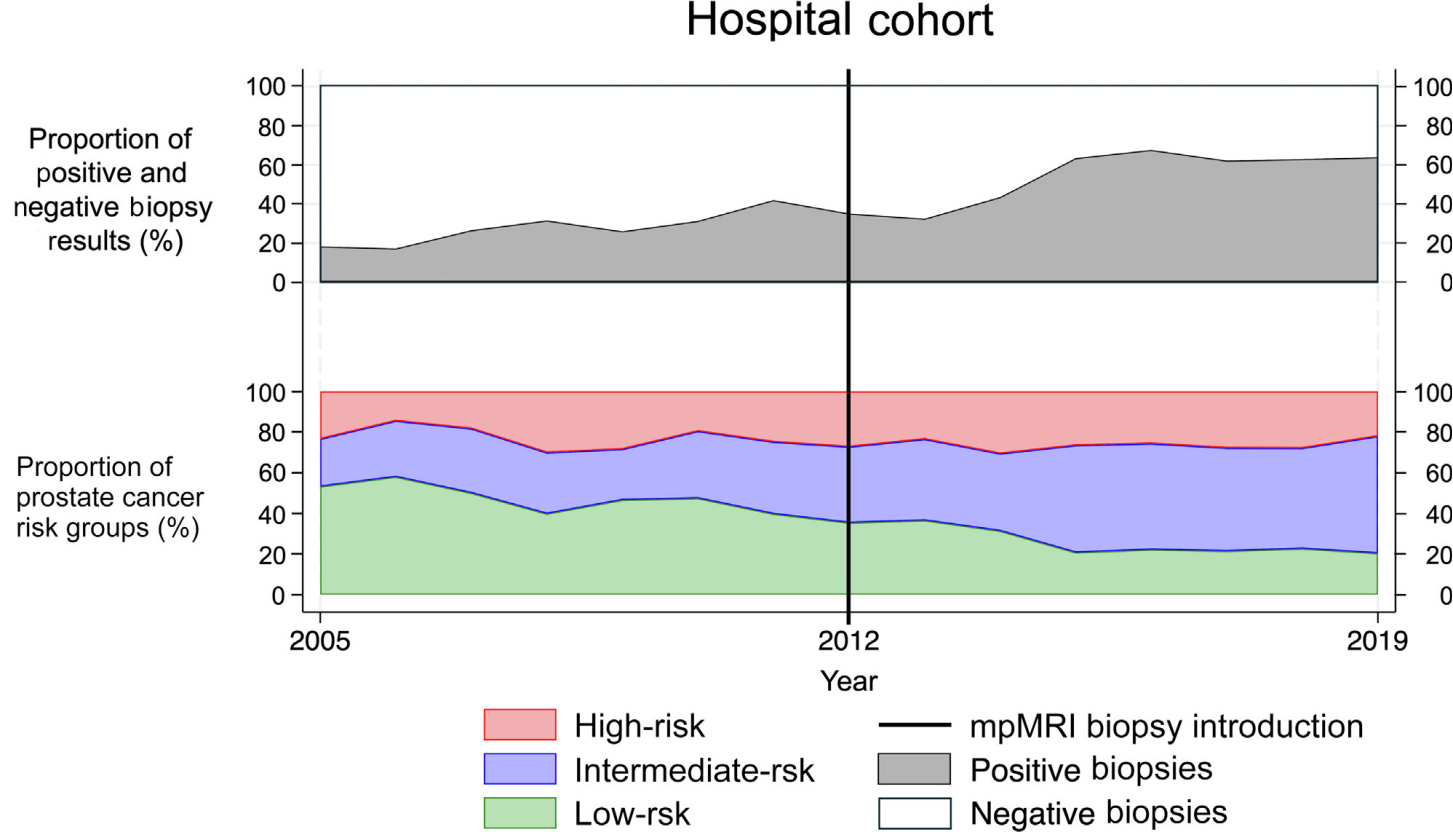
Diagram displaying the results of prostate biopsies from the hospital dataset for each year from 2005 to 2019. It shows the proportion of negative results (white area, top section) and positive results (gray area, top section) and the distribution of risk groups among PCa cases: LR (green area), IR (blue area), and HR (red area). The introduction of mpMRI-guided biopsies is marked (black vertical line) on the timeline in 2012. HR = high risk; IR = intermediate risk; LR = low risk; mpMRI = multiparametric magnetic resonance imaging; PCa = prostate cancer.(For interpretation of the references to colour in this figure legend, the reader is referred to the web version of this article.)

The hospital introduced mpMRI-guided fusion biopsies in 2012. Of all biopsies, 2248 (46.0%) were performed before 2012, whereas 2642 (54.0%) were performed after uptake of mpMRI biopsies. The number of cores and the biopsy method were not standardized in either period. Before the availability of mpMRI, standard TRUS-guided biopsies with either eight or 12 cores were performed via the transrectal approach (median extracted cores: 8 [IQR 8–12]). After the introduction of an mpMRI-guided biopsy, most biopsies were performed using the transperineal approach with real-time fusion of TRUS and the mpMRI image (BiopSee, Innomedicus, Switzerland). In most cases, targeted biopsies were taken (three per lesion and three perilesional) in addition to the 12-core systematic biopsy. Furthermore, some patients in the hospital dataset received saturation biopsies to check eligibility for high-intensity focused ultrasound treatment. The median number of extracted cores after the introduction of mpMRI of the prostate was 21 (IQR: 12–40). Before mpMRI usage (from 2005 to 2011), 72.6% (95% CI: 70.7–74.4%) of biopsies were found to be PCa negative. Among the detected PCa cases, 46.4% (95% CI: 42.4–50.3%) were classified as LR, 30.5% (95% CI: 27.0–34.2%) as IR, and 23.2% (95% CI: 20.0–26.7%) as HR. After mpMRI implementation from 2012 to 2019, 45.7% (95% CI: 43.9–47.7%) of prostate biopsies were PCa negative. Among the positive PCa cases, 24.6% (95% CI: 22.4–26.9%) were LR, 49.0% (95% CI: 46.4–51.6%) IR, and 26.5% (95% CI: 24.2–28.8%) HR PCa. The proportion of negative biopsies as well as the risk group distribution for each year is displayed in Fig. 1.

Overall, 39 out of 62 contacted urological departments completed the mpMRI uptake survey (response rate of 66.1%). The response rate varied between regions of the 13 cantonal cancer registries. Six regions achieved a response rate of ≥75%, while seven regions had a response rate of ≤50%. Among the regions with a response rate of ≤50%, three regions did not provide any responses. The first institutions began introducing mpMRI-guided biopsies in their clinics in 2008. At the end of our study period, 97.1% of biopsies were performed after prior mpMRI.

**Fig. 2:**
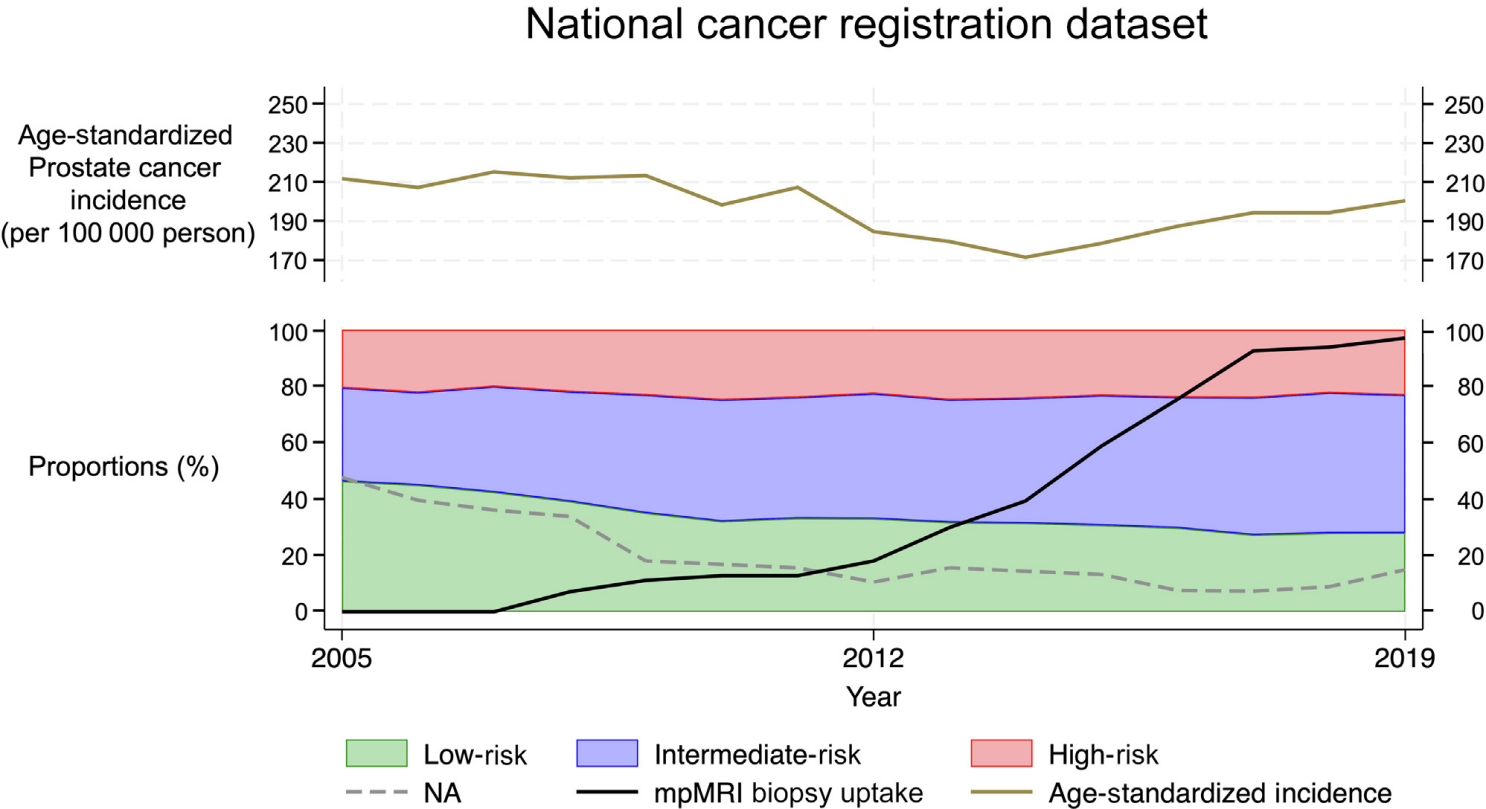
Diagram displaying the results of the NACR dataset. The graph shows the age-standardized PCa incidence (brown, top section), the distribution of risk groups among PCa cases, and the uptake of mpMRI-guided biopsy according to our survey (bottom section, black line). The proportions of LR (blue area), IR (green area), and HR (red area) are displayed between the years 2005 and 2019. Furthermore, the proportions of missing grade information (gray, dashed line) are shown. HR = high risk; IR = intermediate risk; LR = low risk; mpMRI = multiparametric magnetic resonance imaging; NA = no data available; NACR = National Agency for Cancer Registration; PCa = prostate cancer.(For interpretation of the references to colour in this figure legend, the reader is referred to the web version of this article.)

In 2005, the NACR dataset consisted of 3448 PCa cases in 14 cantons, of which 1644 (47.7%) had no data on grade (grade N/A). Among the ones with a reported grade, 836 (46.3%) were LR, 597 (33.1%) were IR, and 371 (20.6%) were HR. In 2019, of 6868 cases registered from 23 cantons, 1026 (14.9%) had missing grade information. Among cases with a registered grade, 1636 (28.0%) were LR, 2853 (48.9%) were IR, and 1353 (23.2%) were HR. The age-standardized PCa incidence as well as the risk group distribution is displayed along the mpMRI uptake for each year in Fig. 2. The Spearman correlation coefficient between the utilization of mpMRI and the proportion of LR PCa cases was –0.976 (*p* < 0.001), IR PCa was 0.981 (*p* < 0.001), and HR PCa was 0.499 (*p* = 0.060).

**Fig. 3:**
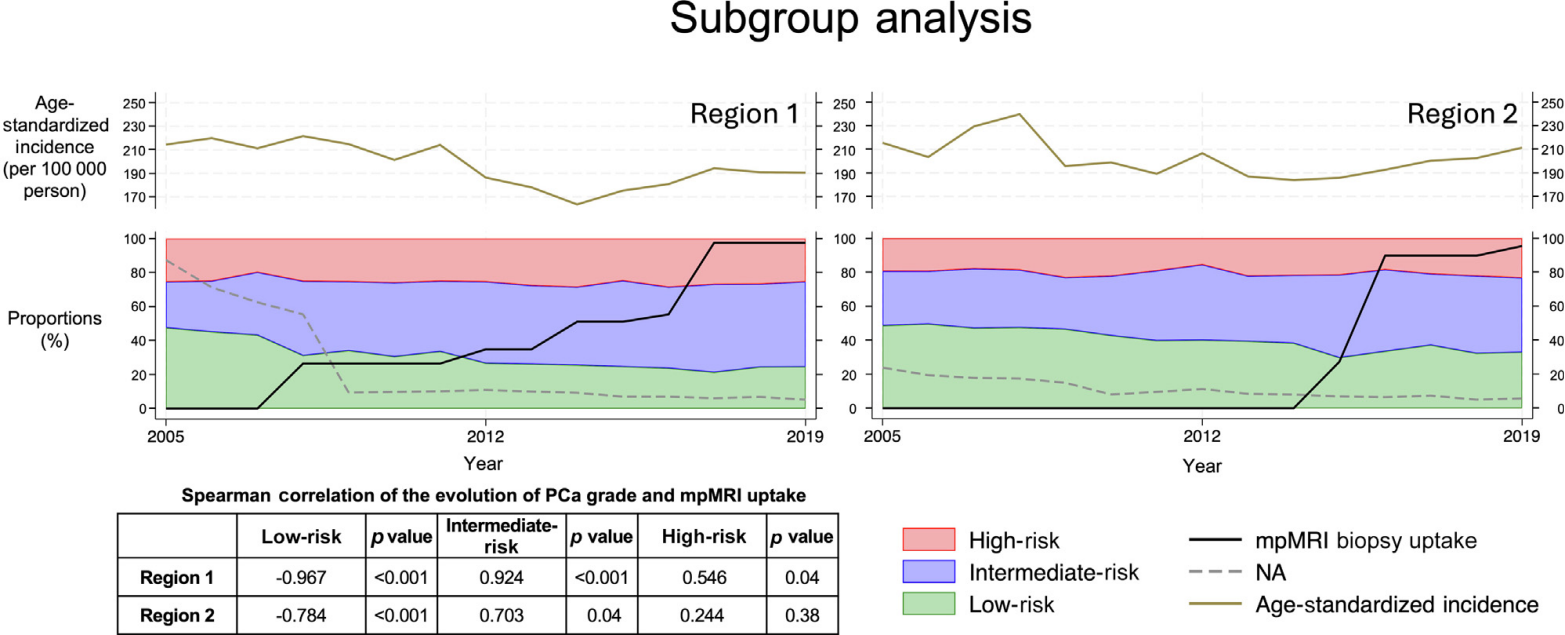
Graphs displaying the results of the subgroup analysis of the NACR dataset. For regions 1 and 2, the graphs show the age-standardized PCa incidence (brown, top section), the uptake of mpMRI-guided biopsy (black, bottom section), and the proportions of missing grade information (gray, dashed line, bottom section). The proportions of LR (blue), IR (green), and HR (red) PCa cases are displayed for each year. Additionally, a table with the Spearman correlation coefficient between mpMRI and the various risk groups over the years is displayed (region 1: Cantons of Zürich and Zug; region 2: Cantons of St. Gallen, Thurgau, and Appenzell Innerrhoden and Ausserrhoden). HR = high risk; IR = intermediate risk; LR = low risk; mpMRI = multiparametric magnetic resonance imaging; NA = no data available; NACR = National Agency for Cancer Registration; PCa = prostate cancer.(For interpretation of the references to colour in this figure legend, the reader is referred to the web version of this article.)

For the regional subgroup analysis (illustrated in Fig. 3), 11 cancer registries were excluded due to data limitations: five did not record data for the entire study period, an additional five had a survey response rate in corresponding urology departments of ≤50%, and one registry had only one answer. Consequently, data from two cantonal registries, the registry from Zürich and Zug (region 1), and the registry from St. Gallen, Thurgau, Appenzell Ausserrhoden, and Appenzell Innerrhoden (region 2), were compared in the subgroup analysis.

**Supplementary Fig. 1:**
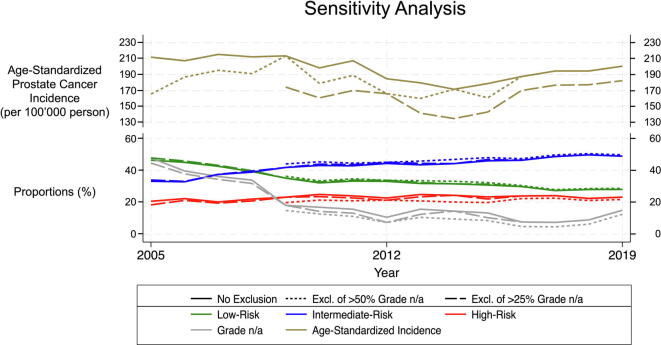
Diagram displaying the sensitivity analysis of the NACR dataset. The graph shows the age-standardized PCa incidence (brown, top section) and the distribution of risk groups among PCa cases. The proportions of LR (blue), IR (green), HR (red) and the proportion of missing grade information (grey) are displayed for each year. For each variable, the solid line displays no exclusion, the dotted line indicates the results with exclusion of cancer registries with ≥25% missing data on grade and the dashed line shows the results with exclusion of cancer registries with ≥ 50% missing data.

The influence of missing data on the distribution of risk groups is depicted in Supplementary Fig. 1.

## 4 Discussion

During the implementation period of an mpMRI-guided prostate biopsy in Switzerland, our study indicates a decrease in LR and an increase in IR PCa cases, while the proportion of HR PCa cases remained largely stable. In the NACR dataset, LR PCa cases comprised initially 46.3% of the diagnosed PCa cases and decreased to 28.0% by the end of the period, while the proportion of IR cases increased from 33.1% to 48.9%. The proportion of HR cases rose slightly from 20.6% to 23.2%. A similar trend was observed in the hospital data, where 53.2% of the detected PCa cases were LR in 2005 and decreased to 20.3% by 2019, while the proportion of IR cases increased from 23.4% to 57.6%. The proportion of HR cases decreased slightly from 23.4% to 22.0%. These changes in the distribution of detected PCa grades were correlated with the introduction of mpMRI in the diagnostic workup for a prostate biopsy in both datasets.

Two large randomized controlled trial assessed the effect of mpMRI-guided biopsies compared with systematic biopsies [3], [4]. In the TRUS control arm of the STHLM3 trial, 41% of the detected PCa cases were LR and 47% IR, compared with 18% LR and 66% IR in the mpMRI arm [4]. In the control arm of the PRECISION study, the PCa cases consisted of 46% LR and 45% IR cases, whereas in the mpMRI arm, 19% were LR and 59% were IR PCa cases [3]. Furthermore, the PROMIS trial reported 18% more “significant PCa” (ISUP ≥2 or a cancer involvement of ≥6 mm in the biopsy) and 5% less clinically “insignificant PCa” cases in their prospective matched mpMRI cohort than in the TRUS cohort [8]. A retrospective population-based cohort study from Sweden demonstrated a population-level decrease in the detection of PCa cases with GS 6 (odds ratio [OR] 0.47, 95% CI: 0.33–0.64) and an increase in PCa cases with GS ≥7 (OR 1.24, 95% CI: 1.02–1.50) with the usage of mpMRI prior to prostate biopsies, compared with when mpMRI was not used [9]. In summary, the studies above showed similar changes in the distribution of PCa risk groups in the mpMRI biopsy pathway to those we observed in our study.

In our hospital dataset, mpMRI and consequently fusion biopsies were introduced in 2012. When analyzing the distribution of risk groups in the hospital data over time, a slight decrease in the frequency of LR cases accompanied by an increase in IR cases was already detectable prior to mpMRI introduction. However, this trend gained significant momentum 2 yr after mpMRI introduction. Possible explanations for this time delay includes the time required to establish confidence in the diagnostic accuracy of mpMRI, which might lead to avoidance of biopsies in patients with low probability for PCa. Further, although mpMRI-guided biopsies became available in 2012, not every biopsy was performed with prior mpMRI or with the use of fusion software, as the decision to employ these methods was left to the treating physician. Additionally, the learning curve of an mpMRI-targeted biopsy could have also played a role. It is known that PCa detection rate of urologists using mpMRI-targeted biopsies increases over an observed period [10], [11]. Furthermore, a repeat biopsy after a PCa-negative biopsy was the standard of care for a TRUS biopsy and therefore was often performed at the beginning of the study period. These multiple random sampling biopsies led to a high proportion of both PCa-negative biopsies and frequently detected low-risk PCa [12], [13], [14].

In the hospital dataset, a significant decrease in the rate of PCa-negative biopsies was observed during the analyzed period. Initially, in 2005, 81.7% of all performed prostate biopsies were PCa negative, which dropped to 36.1% by the end of the study period. The overall rate of PCa-negative biopsies using conventional TRUS-guided biopsies was higher in our dataset than in randomized controlled trials. However, the proportion of PCa-negative biopsies using mpMRI-guided biopsies was comparable with the results of the randomized controlled trials. The STHLM3 trial reported a decrease of PCa-negative biopsies from 59% to 31%, whereas the PRECISION group observed a reduction from 48% to 32% [3], [4]. This reduction in PCa-negative biopsies potentially reduces health care costs by avoiding unnecessary procedures. While specific cost-reduction data are lacking, it could be demonstrated that risk-based screening followed by MRI testing represents a more cost-effective strategy than no screening [15], [16].

According to the results of our survey, national mpMRI uptake prior to a biopsy began in 2008 and surpassed 95% by 2019, which correlated strongly with an increase in IR and a decrease in LR PCa cases. Despite limitations in registration and survey response rates, the subgroup and sensitivity analyses confirmed these findings. The urological departments of region 2 reported a later mpMRI uptake than the departments in region 1, and consequently region 2 showed a later decrease of LR and increase of IR PCa cases (Fig. 3). The sensitivity analysis demonstrated that exclusion of registries with a high number of missing data on PCa grade hardly influenced the distribution of risk groups, but had a larger effect on the age-standardized incidence rate (Supplementary Fig. 1).

The interpretation of the observed shift, with fewer LR and more IR cases, is challenging, and the implications for patient care remain unknown. The substantial decrease in negative biopsies leads to fewer adverse events in patients who underwent a prostate biopsy without a PCa diagnosis [3], [4], [8]. Furthermore, the utilization of mpMRI before deciding on a potential biopsy leads to exclusion of patients with a very low risk of PCa from undergoing a biopsy [3], [4], [8]. However, the presence of a target for the biopsy leads to a substantial increase in the probability of sampling a PCa lesion centrally, thereby increasing the risk of sampling a higher Gleason grade pattern and leading to an increase in ISUP classification and, consequently, a recommendation for active treatment [17], [18]. This problem is accentuated by the fact that a target is often sampled multiple times [19], [20], [21]. Notably, the ProtecT trial demonstrated that, even using conventional TRUS biopsy methods, a large proportion of PCa patients carry a substantial risk of overtreatment [22], [23]. However, the European Association of Urology (EAU) and American Urological Association guidelines do not offer a separate treatment algorithm for PCa based on the biopsy method [1], [24]. This would especially seem relevant when determining eligibility for Active Surveillance, which is the preferred treatment for patients diagnosed with LR PCa [1]. Additionally, patients with favorable IR PCa can be offered Active Surveillance [1]. Favorable is defined in the EAU guidelines based on the histological ratio of the Gleason 4 to Gleason 3 pattern, the number of positive biopsy cores, the tumor length of the biopsy core, and the PSA value [1]. Except for the PSA value, these criteria are all heavily affected by the sampling strategy [25]. Hence, the treating physicians have to be aware of the potentially increased risk of overtreatment. Furthermore, the diagnostic sampling strategy should be included in the decision-making process to determine the most suitable therapy, especially for assessing eligibility for active surveillance.

Our study has some limitations. Swiss cantonal cancer registries record a composite GS, in which the GS of the biopsy is overwritten in case of a prostatectomy by the GS of the prostatectomy specimen. Hence, up- or downstaging after prostatectomy changed the GS in the NACR dataset. In the hospital dataset, GS of the histology of the prostate biopsy was used. The survey was sent to urology departments, resulting in the exclusion of smaller private practices the biopsy methods of which were unavailable for an analysis. Additionally, there might be an overestimation of the adoption of mpMRI technology, as departments with an interest to mpMRI might have been more inclined to participate in the survey. In Switzerland, patients of a region do not necessarily have to be treated at hospitals of the same area, but they are free to seek treatment in another region in Switzerland. Unfortunately, the NACR does not receive information on the region of the treatment, instead on the place of residence. Lastly, throughout the observed period, the recommendations for PSA screening varied, and the biopsy decision-making process was based on individualized patient-doctor discussions.

## 5 Conclusions

The adoption of an mpMRI-guided biopsy within our hospital dataset was paralleled by a reduction in negative prostate biopsies, as was to be expected based on the results of prior randomized controlled trials. Additionally, the analysis of both the hospital and the national data revealed a shift in the distribution of the detected PCa grades following the implementation of mpMRI. Specifically, there was an increase in the proportion of IR cases, while LR cases decreased over time. However, the proportion of HR cases remained relatively unchanged. Whether these changes in the distribution are due to better risk stratification prior to a biopsy, or due to a grade migration effect because of a different diagnostic approach remains unclear and must be the focus of further studies.

  ***Author contributions*:** Thomas Paul Scherer had full access to all the data in the study and takes responsibility for the integrity of the data and the accuracy of the data analysis.

  *Study concept and design*: Scherer, Rohrmann, Poyet.

*Acquisition of data*: Scherer.

*Analysis and interpretation of data*: Scherer, Menges.

*Drafting of the manuscript*: Scherer, Menges, Bieri.

*Critical revision of the manuscript for important intellectual content*: Wildisen, Staehelin, Eberli, Rohrmann, Poyet.

*Statistical analysis*: Scherer, Menges.

*Obtaining funding*: None.

*Administrative, technical, or material support*: None.

*Supervision*: Eberli, Rohrmann, Poyet.

*Other*: None.

  ***Financial disclosures:*** Thomas Paul Scherer certifies that all conflicts of interest, including specific financial interests and relationships and affiliations relevant to the subject matter or materials discussed in the manuscript (eg, employment/affiliation, grants or funding, consultancies, honoraria, stock ownership or options, expert testimony, royalties, or patents filed, received, or pending), are the following: None.

  ***Funding/Support and role of the sponsor*:** None.

  ***Acknowledgments*:** We would like to thank the Cantonal Cancer Registries and the Childhood Cancer Registry for the registration of the data used in this study, namely: Bergeron Y (CR-FR), Bordoni A (CR-TI), Curjuric I, Adam M (CR-AG), Defossez G (CR-VD), Diebold J (CR-LU/UR/OW/NW), Erny S (CR-BS/BL), Konzelmann I (CR-VS), Kuehni C (ChCR), Maspoli M, Bulliard JL (CR-NE/JU), Mousavi M (CR-SG/TG/AI/AR), Perren A (CR-BE/SO), Rapiti E (CR-GE), Rohrmann S (CR-ZH/ZG/SH/SZ), and von Moos R (CR-GR/GL). We also acknowledge the National Agency for Cancer Registration (NACR) for merging the cantonal data and providing the national data, which enabled the national analysis.
