# Supplementary material for: Impact of Prebiopsy Multiparametric Magnetic Resonance Imaging on Prostate Cancer Detection in Switzerland
Source: Eur Urol Open Sci. 2025 Jan 24;73:1–7. doi: 10.1016/j.euros.2025.01.004 (PMC11804726; doi:10.1016/j.euros.2025.01.004)
Supplement: Supplementary Data 1 [file mmc1.docx]

| **Year** | **2005** | **2006** | **2007** | **2008** | **2009** | **2010** | **2011** | **2012** | **2013** | **2014** | **2015** | **2016** | **2017** | **2018** | **2019** | **Total** |
| --- | --- | --- | --- | --- | --- | --- | --- | --- | --- | --- | --- | --- | --- | --- | --- | --- |
| **Biopsies,n** | 257 | 402 | 309 | 295 | 339 | 310 | 336 | 282 | 355 | 308 | 350 | 340 | 428 | 302 | 277 | 4890 |
| **Age,** years  Median  (IQR) | 63.6  (59.2,67.5)  n=257 | 63.1 (58.1,68.7) n=402 | 64.8 (59.5,69.4) n=309 | 64.1 (58.1,69.3) n=295 | 62.8 (57.7,67.9) n=339 | 64.5 (59.0,69.1) n=310 | 63.8 (59.1,68.4) n=336 | 64.0 (57.4,68.3) n=282 | 64.9 (59.7,68.9) n=355 | 65.6 (60.0,70.6) n=308 | 65.8 (59.9,71.1) n=350 | 65.7 (60.2,70.3) n=340 | 65.9 (60.1,71.4) n=428 | 65.9 (59.7,71.3) n=302 | 66.3 (60.5,71.9) n=277 | 64.7 (59.1,69.7) n=4890 |
| **PSA,** ng/ml  Median  (IQR) | 5.0  (3.4,7.7)  n=254 | 4.7  (3.2, 7.2) n=400 | 5.3  (3.5, 8.6) n=290 | 5.0  (3.4, 8.0) n=284 | 4.9  (3.4, 7.6) n=335 | 5.2  (3.5, 8.0) n=307 | 5.1  (3.3, 8.9) (n=334) | 5.5  (3.7, 8.3) n=277 | 5.9  (3.7,10.0)  n=348 | 6.2 (4.2,10.0)  n=291 | 6.1 (4.0,9.5) n=342 | 6.4 (3.7,9.6) n=333 | 5.8 (3.8,9.8) n=407 | 6.6 (4.0,10.9) n=293 | 6.0 (3.4,9.0) n=276 | 5.5 (3.6,8.9)  n=4771 |
| **Pr. Vol,** ml  Median  (IQR) | 37  (29,47)  n=205 | 40  (30,50)  n=331 | 40  (30,60)  n=157 | 40  (27,60)  n=158 | 40.0  (30,55)  n=293 | 45  (30,60)  n=271 | 40  (30,54)  n=308 | 40  (30,55)  n=235 | 42  (30,60)  n=309 | 40  (30,55) n=255 | 40  (30,55)  n=314 | 42  (30,58)  n=320 | 40  (30,56)  n=380 | 45  (32,60)  n=268 | 44  (32,63)  n=254 | 40  (30,56)  n=4058 |

**Supplementary Table 1:** Clinical characteristics of men undergoing prostate biopsy in the hospital dataset. Abbreviations: IQR: Interquartile range, Pr. Vol.: Prostate Volume, PSA: Prostate Specific Antigen.

| **Year** | **2005** | **2006** | **2007** | **2008** | **2009** | **2010** | **2011** | **2012** | **2013** | **2014** | **2015** | **2016** | **2017** | **2018** | **2019** | **Total** |
| --- | --- | --- | --- | --- | --- | --- | --- | --- | --- | --- | --- | --- | --- | --- | --- | --- |
| **Diagnoses, n** | 3448 | 3654 | 3908 | 3925 | 4058 | 4163 | 4632 | 4403 | 4849 | 5521 | 5911 | 6365 | 6766 | 6276 | 6868 | 74747 |
| **<50 years** | 30  (0.9%) | 37  (1.0%) | 50  (1.3%) | 38  (1.0%) | 37  (0.9%) | 50  (1.2%) | 41  (0.9%) | 52  (1.2%) | 52  (1.1%) | 56  (1.0%) | 54  (0.9%) | 55  (0.9%) | 69  (1.0%) | 42  (0.7%) | 44  (0.6%) | 707  (0.9%) |
| **50-54 years** | 107  (3.1%) | 117  (3.2%) | 119  (3.0%) | 142  (3.6%) | 142  (3.5%) | 132  (3.2%) | 180  (3.9%) | 198  (4.5%) | 172  (3.5%) | 191  (3.5%) | 208  (3.5%) | 242  (3.8%) | 242  (3.6%) | 196  (3.1%) | 227  (3.3%) | 2615 (3.5%) |
| **55-59 years** | 296  (8.6%) | 353  (9.7%) | 355  (9.1%) | 316  (8.1%) | 348  (8.6%) | 374  (9.0%) | 420  (9.1%) | 411  (9.3%) | 394  (8.1%) | 494  (8.9%) | 497  (8.4%) | 531  (8.3%) | 562  (8.3%) | 550  (8.8%) | 554  (8.1%) | 6455 (8.6%) |
| **60-64 years** | 625 (18.1%) | 638 (17.5%) | 715 (18.3%) | 709 (18.1%) | 721 (17.8%) | 688 (16.5%) | 811 (17.5%) | 680 (15.4%) | 785 (16.2%) | 850 (15.4%) | 862 (14.6%) | 879 (13.8%) | 1059 (15.7%) | 903 (14.4%) | 1002 (14.6%) | 11927 (16.0%) |
| **65-69 years** | 618 (17.9%) | 699 (19.1%) | 806 (20.6%) | 799 (20.4%) | 876 (21.6%) | 994 (23.9%) | 1024 (22.1%) | 998 (22.7%) | 1109 (22.9%) | 1145 (20.7%) | 1264 (21.4%) | 1429 (22.5%) | 1350 (20.0%) | 1250 (19.9%) | 1355 (19.7%) | 15716 (21.0%) |
| **70-74 years** | 672 (19.5%) | 695 (19.0%) | 704 (18.0%) | 760 (19.4%) | 797 (19.6%) | 736 (17.7%) | 850 (18.4%) | 786 (17.9%) | 917 (18.9%) | 1086 (19.7%) | 1263 (21.4%) | 1421 (22.3%) | 1570 (23.2%) | 1437 (22.9%) | 1474 (21.5%) | 15168 (20.3%) |
| **75-79 years** | 491 (14.2%) | 492 (13.5%) | 559 (14.3%) | 504 (12.8%) | 525 (12.9%) | 538 (12.9%) | 613 (13.2%) | 577 (13.1%) | 570 (11.8%) | 708 (12.8%) | 756 (12.8%) | 823 (12.9%) | 886 (13.1%) | 917 (14.6%) | 1123 (16.4%) | 10082 (13.5%) |
| **80-84 years** | 357 (10.4%) | 382 (10.5%) | 352  (9.0%) | 384  (9.8%) | 346  (8.5%) | 359  (8.6%) | 399  8.6%) | 384  (8.7%) | 473  (9.8%) | 515  (9.3%) | 567  (9.6%) | 536  (8.4%) | 555  (8.2%) | 549  (8.7%) | 592  (8.6%) | 6750 (9.0%) |
| **85+ years** | 252  (7.3%) | 241  (6.6%) | 248  (6.3%) | 273  (7.0%) | 266  (6.6%) | 292  (7.0%) | 294  (6.3%) | 317  (7.2%) | 377  (7.8%) | 476  (8.6%) | 440  (7.4%) | 449  (7.1%) | 473  (7.0%) | 432  (6.9%) | 497  (7.2%) | 5327 (7.1%) |
| **PSA,**  ng/ml  Median(IQR) | 12.0  (6.2,36.7)  n=1527 | 10.4 (6.3,28.0)  n=1863 | 9.6  (6.0,22.0)  n=2107 | 9.9  (6.0,22.0)  n=2266 | 9.0  (6.0,20.0)  n=2766 | 9.1  (5.8,21.0)  n=2912 | 8.8 (5.7,18.5)  n=3246 | 9.0 (5.8,22.5)  n=3217 | 9.0 (5.7,22.8)  n=3311 | 9.1 (5.8,23.1)  n=3674 | 9.4 (5.9,23.4)  n=4126 | 9.2 (5.8,20.8)  n=4937 | 9.0 (5.7,21.7)  n=5360 | 9.3 (6.0,23.0)  n=4784 | 10.2 (6.0,47.3)  n=5577 | 9.3  (5.9,23.6) n=51673 |

**Supplementary Table 2:** Clinical characteristics of the prostate cancer patients in the dataset of the National Agency for Cancer Registration. Abbreviations: IQR: Interquartile range, PSA: Prostate Specific Antigen.

| **Year** | **2005** | **2006** | **2007** | **2008** | **2009** | **2010** | **2011** | **2012** | **2013** | **2014** | **2015** | **2016** | **2017** | **2018** | **2019** |
| --- | --- | --- | --- | --- | --- | --- | --- | --- | --- | --- | --- | --- | --- | --- | --- |
| **Total Biopsies, n** | 257 | 402 | 309 | 295 | 339 | 310 | 336 | 282 | 355 | 308 | 350 | 340 | 428 | 302 | 277 |
| **Negative Biopsy, n** | 210 (81.7%) | 333 (82.8%) | 227 (73.5%) | 202 (68.5%) | 251 (74.0%) | 213 (68.7%) | 195 (58.0%) | 183 (64.9%) | 240 (67.6%) | 174 (56.5%) | 128 (36.6%) | 110 (32.4%) | 162 (37.9%) | 112 (37.1%) | 100 (36.1%) |
| **Positive**  **Biopsy, n** | 47  (18.3%) | 69  (17.2%) | 82  (26.5%) | 93  (31.5%) | 88  (26.0%) | 97  (31.3%) | 141 (42.0%) | 99  (35.1%) | 115 (32.4%) | 134 (43.5%) | 222 (63.4%) | 230 (67.6%) | 266 (62.1%) | 190 (62.9%) | 177 (63.9%) |
| **low-risk, n** | 25  (53.2%) | 40  (58.0%) | 41  (50.0%) | 37  (39.8%) | 41  (46.6%) | 46  (47.4%) | 56  (39.7%) | 35  (35.4%) | 42  (36.5%) | 42  (31.3%) | 46  (20.7%) | 51  (22.2%) | 57  (21.4%) | 43  (22.6%) | 36  (20.3%) |
| **intermediate-risk, n** | 11  (23.4%) | 19  (27.5%) | 26  (31.7%) | 28  (30.1%) | 22  (25.0%) | 32  (33.0%) | 50  (35.5%) | 37  (37.4%) | 46  (40.0%) | 51  (38.1%) | 117 (52.7%) | 120 (52.2%) | 135 (50.8%) | 94  (49.5%) | 102 (57.6%) |
| **high-risk, n** | 11  (23.4%) | 10  (14.5%) | 15  (18.3%) | 28  (30.1%) | 25  (28.4%) | 19  (19.6%) | 35  (24.8%) | 27  (27.3%) | 27  (23.5%) | 41  (30.6%) | 59  (26.6%) | 59  (25.7%) | 74  (27.8%) | 53  (27.9%) | 39  (22.0%) |

**Supplementary Table 3:** Results of prostate biopsies from the hospital dataset for each year from 2005 to 2019 displayed in Figure 1.
